# Modelling the significance of celebrity endorsement and consumer interest on attitude, purchase intention, and willingness to pay a premium price for green skincare products

**DOI:** 10.1016/j.heliyon.2023.e16765

**Published:** 2023-05-26

**Authors:** Abdullah Al Mamun, Farzana Naznen, Qing Yang, Mohd Helmi Ali, Nik Mohd Hazrul Nik Hashim

**Affiliations:** a UKM - Graduate School of Business, Universiti Kebangsaan Malaysia, 43600, UKM Bangi, Selangor Darul Ehsan, Malaysia; b UCSI Graduate Business School, UCSI University, Cheras, 56000 Kuala Lumpur, Malaysia

**Keywords:** Celebrity endorsement, Attitude towards advertisement, Attitude towards brands, Purchase intention, Willingness to pay premium price, Green skincare products

## Abstract

Due to the major shift in online purchasing during the COVID-19 lockdown, celebrity endorsement marketing has gained traction. Concurrently, COVID-19 has also transformed consumers' attitudes toward using eco-friendly products, such as green skincare products, to ensure a healthier lifestyle. This study developed a comprehensive framework based on the stimuli-organism-response theory and the parasocial interaction theory to empirically evaluate the impact of celebrities' credibility attributes and consumers' interests in celebrities on their attitudes towards advertisements for endorsed green skincare products, their intentions to make a purchase, and their willingness to pay a premium price for these products. 778 Malaysian consumers participated in the online survey, and their responses were analyzed using partial least squares structural equation modelling (PLS-SEM). The results showed the positive effects of credibility traits (trustworthiness – β = 0.100, p-value = 0.026; exquisite personality – β = 0.075, p-value = 0.028; dignified image – β = 0.152, p-value = 0.001; expertise – β = 0.221, p-value <0.001), and customer attention to celebrities (β = 0.184, p-value <0.001) on their attitudes toward endorsed advertisements. Likewise, credibility features (exquisite personality – β = 0.116, p-value = 0.002; dignified image – β = 0.112, p-value = 0.017; expertise – β = 0.207, p-value <0.001) and customers' companionship with celebrities (β = 0.142, p-value = 0.001) also have a significant positive impact on attitudes towards brands. Finally, consumers' purchasing intentions and willingness to pay premium prices for green skincare products were strongly influenced by their attitude toward advertising (β = 0.484, p-value <0.001) and brands (β = 0.326, p-value <0.001). Evidently, the findings of this study may help players in the cosmetics industry enhance their marketing and promotion tactics for eco-friendly beauty and personal care products.

## Highlights

• Celebrity endorsers credibility has a positive effect on attitude towards green skincare product advertisements.
• Exquisite personality, image, expertise and customer's interest has a positive effect on attitude towards green skincare product brands.
• Consumers attitude towards advertisement and green skincare product brands has a significant effect on consumers' purchase intention.
• Consumers' purchase intention and willingness to pay premium price for green skincare products.

## 1 Introduction

The global COVID-19 pandemic has significantly affected both developed and emerging markets, especially the commercial activities, due to various pandemic restrictions that include social distancing and lockdowns [1]. Recent studies have also discussed how the COVID-19 pandemic has clearly affected earnings, supply of goods, and product accessibility, which subsequently influence consumers’ purchasing behaviour [2]. These changes have caused numerous economic challenges for businesses worldwide, resulting in the needs to adapt and innovate for their business sustainability in the market. Substantial changes in marketing and promotion strategies have become necessary to address the business downturn.

During the lockdown period of COVID-19, people mostly stayed at home and had plenty of personal time, which significantly increased individuals' interest in personal skincare and aesthetic trends, notably green cosmetics and skincare [3]. In contrast, worldwide, the beauty sector has seen declines in revenue of 20–30% in the year 2020 as a result of the global pandemic [4]. Additional findings from the survey by Mckinsey showed that customers in the UK (48%), US (36%), and Japan (24%), spent less money on beauty products during COVID-19 [4]. Reports also indicated a 15% decrease in purchases of skincare and cosmetics in India [5]. Malaysian authorities have reportedly dealt with a similar situation with the trade of beauty and personal care (B&PC) products. According to the United Nations Comtrade database [6], Malaysia imported B&PC items from around the world worth USD 1.2 billion in 2019. However, that amount dropped by 16.67% in 2020. Malaysia's natural cosmetics market is projected to expand annually by 3.46%, although revenue in 2022 only amounts to US$47.38 million, with a rise of only $0.64 million from 2021 [7]. In light of the facts, it is clear that the natural and green B&PC product in Malaysia experienced a considerable revenue and sales gap during the time of the COVID-19 pandemic. As a consequence of this, it is essential to conduct research into the aspects that may strengthen marketing and promotion strategies through celebrity endorsements in order to keep the green B&PC businesses alive.

Imported cosmetic products, such as beauty and skincare products from the United States, are favourably received and gained positive market reputation among Malaysian consumers, as these products are perceived as high-quality, organic or natural, and vegan without involving animal testing [8]. The COVID-19 pandemic has triggered environmental concerns among people to adopt green lifestyles and green consumption [9]. Green skincare products are formulated from botanically sourced ingredients and contain natural carrier agents; they are free of synthetic chemicals that are beneficial to the skin [10]. However, due to the overstated claims in advertisements, consumers are being found to be sensitive to green products [11]. Concurrently, despite the growing significance of green products, the adoption of green skincare products has yet to be thoroughly researched.

In this highly competitive market, businesses and brands must pursue to establish a solid consumer base [12]. In line with that, celebrity endorsement has been recognised as an effective advertising approach to increase consumer loyalty and interest [13]. Product marketing and endorsement by celebrities and social media influencers may also have a significant impact on millennials' purchasing decisions [14,15]. Celebrity endorsement can be more appealing and increases market penetration, which significantly influence consumers' attitude towards the endorsed products or brands [12]. Prior studies explored the influence of various factors like the credibility of celebrity endorsers [[16], [17], [18], [19]], the interoperability of the celebrity and the product [15], and the congruency of the product and endorsers [[20], [21], [22]]. However, the significance of factors like the dignified image of celebrities and consumers’ attachment to celebrities have received scant attention [12,23].

Studies have noted the positive implications of celebrity endorsement on key marketing criteria, such as more favourable attitude towards advertisements [17,18,24,25], attitude towards brands [21,24], intention to purchase [17,[26], [27], [28]], and brand equity [12]. In addition, customers' willingness to pay a premium price for green products has increased as a result of rising knowledge and awareness over health-related issues and environmental sustainability. A study by SivaKumar and Gunasekaran [29] found that millennials are more willing to pay higher price for environmentally friendly products, even if those products don't meet all of their necessities. However, there is a substantial knowledge gap in regards to green skincare products and cosmetics, which can be due to the ineffective and inefficiency of marketing communication [30]. Furthermore, prior research mostly examined how endorsements affected customers' perceptions and intentions to buy the recommended items, and only a select handful studies were conducted on how endorsements from renowned celebrities may influence consumers' willingness to pay premium price [31]. Most notably, studies exploring the role of celebrity endorsement in marketing activities amid disruptive circumstances are sparse. To the best of the researchers' knowledge, only a handful of studies have examined how celebrity endorsement promotes purchases of eco-friendly skin care products in Malaysia, especially in the context of the economic crisis triggered by the COVID-19 pandemic. Given that customers may not be able to try out a product by visiting outlets directly and would instead rely entirely on referrals from celebrity endorsements, this study should contribute to the body of knowledge in the domain of market research by providing insight into the consumers' behavioural intentions in such situations.

Recognising the aforementioned gaps in literature, the current study aimed to examine the influence of celebrity endorsement on consumers’ attitude and purchase intention to purchase green skincare products endorsed by celebrities, and willingness to pay premium price for these products during a global critical circumstance such a COVID-19. This study also investigated the combined impacts of celebrity endorser credibility and consumer interests as the stimulus, which is a novel contribution to the literature of marketing strategy in green skincare products, particularly in Malaysia. The outcomes of this research should help personnel working in the cosmetics business development to identify better marketing strategies for promoting eco-friendly beauty and personal care products. In addition, the comprehensive framework of this study would let practitioners and future academics to research more on different factors that may help to find effective solutions of issues relating to marketing and pricing of products in unusual situations like the worldwide COVID-19 outbreak.

Following sections of this paper are divided into six parts. Section 1 offers a literature review along with the study's theoretical foundations and assumptions. The second section describes the methods employed, while the third section examines the data analysis and conclusions. In Section 4, the study findings are discussed in further detail. Section 5 covers the theoretical and practical ramifications of the research findings. In section 6, a brief overview, study limitations, and recommendations for further research are presented.

## 2 Literature review

### 2.1 Theoretical background

Mehrabian and Russell [32] introduced the stimuli-organism-response (S–O-R) theory, which was then modified by Jacoby [33]. The theory suggests the effects of environmental elements as stimuli (S) on emotional and cognitive attitudes (O), resulting in behavioural responses (R). This theory has been widely used in various marketing studies, such as studies on advertising [34,35] and celebrity endorsement [19,36,37].

Using the S–O-R framework, the current study attempted to identify key credibility attributes of celebrity endorsers that influence consumers' cognitive and emotional responses, resulting in the formation of favourable attitude towards endorsed green skincare product advertisements and attitude towards endorsed green skincare product brands. Accordingly, “stimuli” refers to a motivating force. Min et al. [34] defined “stimuli” as external factors that affect one's emotional state and subsequently, behaviour. Considering the focus of the current study, “stimuli” was viewed as credibility attributes of celebrity endorsers based on the recommendations by Burnasheva and Suh [19]. Consumers' cognitive and emotional responses are referred to as the “organism” after they are being exposed to stimuli [34]. At this point, consumers form their own views, thoughts, and even attitude towards advertisements, celebrities, or brands [34]. The current study viewed consumers' attitude towards endorsed green skincare product advertisements and attitude towards endorsed green skincare product brands as “organisms”. Lastly, the formed behaviour represents “response” in the S–O-R theory [34]. In this study, “response” was measured as consumers' purchase intention [19,34] and willingness to pay premium price for green skincare products.

Apart from the S–O-R theory, this study considered the parasocial interaction (PSI) theory to examine the influence of consumers’ attention and companionship on their attitude and purchase intention, which was separately evaluated in prior studies [23,38]. Horton and Wohl [39] introduced the PSI theory and described the theory as a one-sided relationship between the media figures and the audience, resulting in a sense of attachment, companionship, and affiliation with the media figures among the audience after being exposed to the media figures often [40]. Celebrity-follower activities on online platforms with universality, active engagement, and various operations emphasise marketing interactivities [38].

### 2.2 Celebrity endorsers’ trustworthiness (ECT)

Yang [41] described trustworthiness of celebrity endorsers (ECT) as perceived integrity, honesty, and believability of celebrity endorsers. The qualities of endorsers' credibility and authenticity are connected to trustworthiness [42] and consumers display a certain level of confidence towards celebrity endorsers [18]. Therefore, endorsers who give the image of consistency, honesty, reliability, and security are employed by businesses and brands to maximise the value of ECT [16]. In a recent study, Punjani and Kumar [43] identified ECT as a significant predictor of consumers' attitude towards advertisements and purchase intention. Consumers perceive a trustworthy and expert celebrity as their measure of quality justification in developing their attitudes towards advertise and product [44]. Meanwhile, Wang et al. [16] and Cespedes-Dominguez et al. [22] presented empirical evidence on the direct and positive influence of ECT on consumers' attitude towards brands. Despite the significance of celebrity endorsement, Yang [41] noted the development of negative attitude towards advertisements and brands among consumers when a celebrity simultaneously endorses multiple products or brands; this raises questions on the celebrity endorser's ethics and trustworthiness from the viewpoints of consumers. In view of the above, the following hypotheses were proposed for testing in the current study:

H1a *Trustworthiness of celebrity endorsers positively influences Malaysian consumers' attitude towards endorsed green skincare product advertisements.*

H1b *Trustworthiness of celebrity endorsers positively influences Malaysian consumers' attitude towards endorsed green skincare product brands.*

### 2.3 Celebrity endorsers’ exquisite personality (ECP)

Exquisite personality (ECP) can be described as a form of attractiveness in terms of physical beauty, personality traits, and elegant lifestyle, which serves as one of the key celebrity attributes that make a specific celebrity endorser appealing to consumers [45]. The growing popularity of employing celebrities as product, service, or brand endorsers has established attractiveness as an essential feature of consideration. Sallam and Wahid [46] identified attractiveness as the most significant attribute of credibility in improving consumers' attitude towards advertisements and brands, as well as purchase intention. Customers who compare attractiveness may be more sensitive to beautiful celebrities who wear luxury brands, implying an interactive relationship between the customer and luxury brand advertisements promoted by trendy celebrities [47]. In another study, Eren-Erdogmus et al. [48] similarly proved that the attractiveness of a celebrity endorser can enhance consumers' attitude towards green advertising. Besides that, Keller [49] noted the integration of attractive personality of highly credible celebrity endorsers with the brand reputations. Pradhan, Duraipandian, and Sethi [26] focused on the context of cosmetics brands and similarly found that attractive celebrity endorsers do positively influence consumers’ attitude towards the endorsed cosmetics brands due to the resemblance of their attractive personality with the brands. Customers who compare attractiveness may be more sensitive to beautiful celebrities who wear luxury brands, implying an interactive relationship between the customer and luxury brand advertisements promoted by trendy celebrities [47]. Based on the empirical evidence and findings presented in earlier studies, the following hypotheses were proposed for testing in the current study:

H2a *Exquisite personality of celebrity endorsers positively influences Malaysian consumers' attitude towards endorsed green skincare product advertisements.*

H2b *Exquisite personality of celebrity endorsers positively influences Malaysian consumers' attitude towards endorsed green skincare product brands.*

### 2.4 Celebrity endorsers’ dignified image (ECD)

As part of the credibility attributes of celebrity endorsers, dignified image (ECD) emphasises the personal and social life of celebrity endorsers and their participation in any social or environmental activities [17]. Moreover, a celebrity endorser's off-screen behaviour can indirectly influence the endorsed product or brand itself [49] and substantially affect consumers' views of the endorsed product or brand [17]. Displaying an image that contradicts the brand or product image causes “vampire effect”, reflecting poor credibility of the celebrity endorser, which is not desirable by any brand or product [41]. In such cases, consumers who pay attention to the image of the appointed celebrity endorsers are less likely to relate themselves to brands or products that are endorsed by controversial celebrity endorsers [50]. For example, a world-renowned tennis player, Maria Sharapova, was found guilty of doping and eventually suspended from Nike and TAG Heuer advertising contracts [51]. However, Lee et al. [52] reported otherwise—the study found limited association between celebrity scandals and consumers' evaluation of the endorsed brands. Based on the findings of prior studies, the current study tested the following hypotheses:

H3a *Dignified image of celebrity endorsers positively influences Malaysian consumers' attitude towards endorsed green skincare product advertisements.*

H3b *Dignified image of celebrity endorsers positively influences Malaysian consumers' attitude towards endorsed green skincare product brands.*

### 2.5 Celebrity endorsers’ expertise (ECE)

Expertise (ECE) refers to one's levels of recognised knowledge, skills, and competencies. An expertise is recognised based on one's capacity to deliver knowledge based on personal competencies or mastery of the field [53]. When it comes to celebrity endorsement, ECE suggests the celebrity endorser's knowledge of the product category and field [27]. A celebrity-endorsed advertisement is more likely to be successful when the celebrity endorser's expertise in the product category and field can convince consumers [18]. A celebrity with specific expertise promotes a credible association with the endorsed product or brand; for example, sports celebrities are considered to have expert judgments on sporting goods [20]. To avoid arousing customers' doubts, Teng et al. [21] recommended that if a celebrity introduces a brand endeavour that is not largely the celebrity's area of specialization, it is better for the celebrity to not even have an executive position. Given the importance of an endorser's competence in shaping customers' perceptions of brands and purchasing intentions, a celebrity endorser with a higher level of perceived expertise is more compelling than an endorser with a low level of competence [16]. A knowledgeable celebrity endorser also successfully attracts consumers purchasing the endorsed products if they consider the celebrity endorser is knowledgeable enough regarding the products [15] and growing positive brand attitude [21]. In view of the above, the following hypotheses were proposed for testing:

H4a *Expertise of celebrity endorsers positively influences Malaysian consumers' attitude towards endorsed green skincare product advertisements.*

H4b *Expertise of celebrity endorsers positively influences Malaysian consumers' attitude towards endorsed green skincare product brands.*

## 3 Consumers’ companionship (CIC)

Consumers are more likely to consider their celebrity-follower partnership when they explore a product or brand and trust that the endorsed product or brand reflects the values of their favourite celebrities [49]. Focusing on the context of conventional mass media, Hung [54] found the relationship between consumers and celebrity endorsers as a factor that positively influences consumers' attitude towards advertisements and purchasing behaviour. Moreover, the follower-celebrity parasocial relationship motivates the readiness of followers to support celebrity-endorsed products [54,55]. Several other prior studies demonstrated the significant and positive influence of parasocial interactions between viewers and TV shopping hosts on consumers’ attitude towards advertisements [56], attention on TV shopping programmes, and purchase intention [57]. Thus, the current study postulated the following hypotheses:

H5a *Consumers' companionship with celebrity endorsers positively influences Malaysian consumers' attitude towards endorsed green skincare product advertisements.*

H5b *Consumers' companionship with celebrity endorsers positively influences Malaysian consumers' attitude towards endorsed green skincare product brands.*

### 3.1 Consumers’ attention (CIA)

A popular media figure attracts attention and reflects credibility attributes that may be transmitted to the endorsed product or brand through various advertising platforms [23]. Consumers’ interests in celebrity-endorsed advertisements promote searching activities [54,58]. In other words, hiring celebrities in advertisements increases engagement actions among consumers [23]. A celebrity endorser gains the attention of consumers, and celebrity endorsement is more likely to stimulate favourable perceptions towards the advertisements and the endorsed product or brand among consumers, resulting in higher purchase intention [59,60]. Thus, the current study proposed the following hypotheses for testing:

H6a *Consumers' attention to celebrity endorsers positively influences Malaysian consumers' attitude towards endorsed green skincare product advertisements.*

H6b *Consumers' attention to celebrity endorsers positively influences Malaysian consumers' attitude towards endorsed green skincare product brands.*

### 3.2 Attitude towards advertisements (ATA)

Favourable attitude towards brands can be measured by consumers' ratings of specific advertisements, such as like ability, disagreeable, and satisfaction [25]. The influence of attitude towards brands on purchase intention has been comprehensively explored in studies across diverse disciplines [43]. Consumers who have favourable mindset towards their ecological concerns tend to respond to green advertisements [61] and purchase green products [62]. Zhang et al. [63] identified the credibility of endorsers as a major factor that influences consumers’ attitude towards green advertising and purchase intention of green products. On a similar note, Bravo and Lee [64] identified attitude towards brands as a significant predictor of purchase intention. However, Kumar and Tripathi [18] reported otherwise; although the study found no direct association between ATA and purchase intention of green products, celebrity endorsement was suggested due to the advantages of having celebrity endorsers in green advertisements. Thus, the current study hypothesised the following:

H7 *Malaysian consumers' favourable attitude towards celebrity-endorsed green skincare product advertisements positively influences their purchase intention.*

### 3.3 Attitude towards celebrity-endorsed green skincare product brands (ATS)

Consumers' attitude towards brands refer to the extent of consumers having faith in the brands' honesty and capacity to deliver their promises [65]. Earlier studies presented numerous evidences on the strong correlation between consumers' brand perceptions and purchasing behaviour [28,52,66]. Therefore, it is pivotal to also explore the influence of celebrity endorsement on consumers' attitude towards brands, as well as how celebrity endorsers can improve the credibility of the endorsed brands and purchase intention [67]. Singh and Banerjee [17] noted that the use of celebrity endorsement favourably influences consumers' brand perceptions and purchase intention, enhancing the popularity of the endorsed brands. In another study, Schmidt et al. [68] examined consumers' responses towards various forms of athletes' activism regarding sports brands and revealed the negative influence of riskier activism on the brands and purchase intention among consumers. In a more recent study that focused on green product endorsement, Kumar and Tripathi [18] revealed the significant influence of consumers’ attitude towards green product brands on their intention to purchase celebrity-endorsed green product brands. Based on the findings of prior studies, the current study proposed the following hypothesis:

H8 *Malaysian consumers' favourable attitude towards celebrity-endorsed green skincare brands positively influences their purchase intention.*

### 3.4 Purchase intention (SCPI)

Purchase intention is a combination of consumers' interest towards a particular product and the likelihood of purchasing the product, which closely reflects consumers' brand or product preference [69]. It also reflects consumers' desire and ability to purchase a particular product [16]. Bagozzi et al. [70] specifically linked purchase intention to one's tendency towards a brand. Purchase intention measures consumers' future contributions towards a particular product or brand [69]. The selling, marketing, and advertising activities of brands positively influence consumers' purchase intention [71]. Most of the prior studies concluded the significance of celebrity endorsement on consumers' attitude and purchase intention [17,27,28]. Celebrity endorsement has been viewed as more genuine or natural; for instance, a celebrity endorser who uses a specific product in the daily routine reflects stronger credibility effect on consumers' purchase intention and even willingness to pay premium price for the endorsed product [53]. Based on the assertions of prior studies, the following hypothesis was proposed for testing in this study:

H9 *Favourable purchase intention positively influences willingness to pay premium price among Malaysian consumers*.

### 3.5 Willingness to pay premium price (WPPP)

A brand gains premium pricing opportunity when there are more consumers who are willing to pay high price for the product than the number of consumers who are willing to pay for the same product from different brands [72]. Customers constantly look for safety and are prepared to pay a higher price for healthier product options [73]. Certain consumers are willing to pay premium price for non-functional benefits that allow them to enjoy hedonic involvement with a particular brand, such as psychological and spiritual gratification [74,75]. Moreover, depending on who is promoting a product, consumers are often willing to pay almost 20% more for the product, resulting in increased revenue for the firm [76]. Earlier studies also presented empirical evidence on the significant influence of source credibility on purchase intention and willingness to pay premium price [53,77,78].

**Fig. 1:**
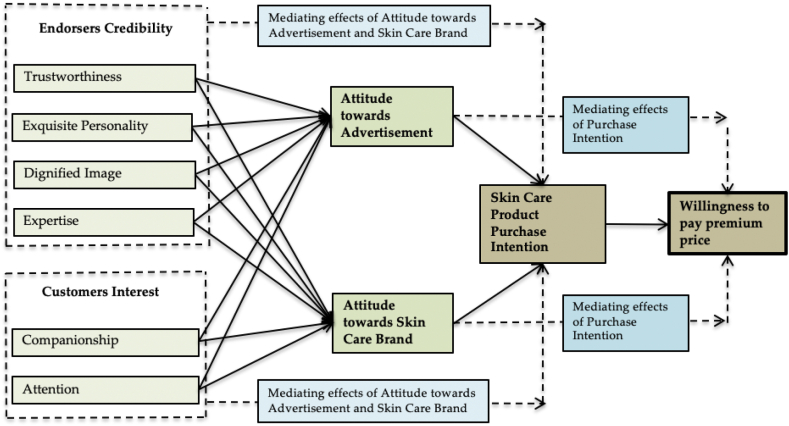
Research framework.

With that, all proposed hypotheses in this study are presented in Fig. 1.

## 4 Methodology

### 4.1 Population and sampling

The target population for this study was young adults aged 18 and older from all demographic groups (i.e., culture, race, education, and occupation) across 14 major states of Malaysia. The respondents were chosen using convenience sampling because it allows researchers to reach respondents who are easily accessible from any region and segment of the entire population [79]. The required sample size was determined using G*Power (version 3.1.9) as recommended by Faul et al. [80]. Setting the parameters in the tool at power of 0.9, effect size of 0.15 (0.15 represent medium effect size accordingly to Cohen [81]), and nine predictors, the calculated sample size for this study was 141. However, this study opted to obtain a larger sample size, almost five times the minimum sample size, to compensate for any potential issues caused by a small sample size.

### 4.2 Data collection

Google Forms was used to conduct an online questionnaire survey. The link to the online survey questionnaire was sent by email or many social networking sites, including Facebook, Instagram, LinkedIn, and WhatsApp. The data was gathered between October 1 and November 30, 2021. A total of 800 acknowledgements of questionnaire distribution were made. Out of those, 778 responses (97.25% of the total distribution) were deemed complete for relevant data analysis. This study has been performed in accordance with the Declaration of Helsinki. Written informed consent for participation was obtained from respondents who participated in the survey.

### 4.3 Instrument

For the development of the current study's questionnaire, validated instruments from prior studies were adopted and adapted with minimal modifications. The questionnaire was developed in a clear and unbiased manner for the respondents to clearly understand the questions. Items that measured ECT, ECP, and ECD were adopted from Singh and Banerjee [17,82]. Meanwhile, five items that measured ECE were adopted from Munnukka et al. [83]. CIC in this study was measured based on items adopted from Ding and Qui [38]. Additionally, five items that measured CIA were adopted from Cheah et al. [23]. On the other hand, ATA and ATS in this study were measured based on items adopted from Munnukka et al. [83] and Singh and Banerjee [17]. Five items were adopted from Rodgers [84] and Barber and Taylor [85] to measure SCPI. Lastly, five items were adopted from Abdeen et al. [86], Godey et al. [87], and Yazdanparast, Joseph, and Muniz [88] to measure WPPP. For this study, the respondents were required to provide their responses according to the five-point Likert scale, with endpoints of “1” (strongly disagree) and “5” (strongly agree) for all variables except for SCPI and WPPP. In order to address the frequent method bias from single-source data, a seven-point Likert scale with the endpoints “1" (strongly disagree) and “7" (strongly agree) was employed to measure SCPI and WPPP [89]. All items used in this study presented in Supplementary Material S1. *Survey Instrument*.

### 4.4 Common method variance (CMV)

Harman's single-factor test is commonly used to address the CMV issue [90]. The results of the single-factor test in this study revealed the highest factor accounting for 31.874%, which did not exceed the recommended maximum limit of 50% [89]. This indicated the insignificant effect of CMV on the study's model. As recommended by Kock [91], a full multicollinearity test was also performed for all components to evaluate common method biasness. Variance inflation factor (VIF) of more than 3.3 reflects the presence of multicollinearity issue [92]. As for the current study, the recorded VIF of all variables did not exceed 3.3 (see Table 1), which confirmed no multicollinearity issue and the absence of bias from the single-source data [91].

**Table 1:** Full collinearity.

	ECT	ECP	ECD	ECE	CIC	CIA	ATA	ATS	SCPI	WPPP
VIF	2.648	1.298	2.428	2.581	1.256	1.462	1.608	1.316	3.127	2.736

**Note:** ECT: Endorsers' Credibility–Trustworthiness; ECP: Endorsers' Credibility–Exquisite Personality; ECD: Endorsers' Credibility–Dignified Image; ECE: Endorsers' Credibility–Expertise; CIC: Customers' Interest–Companionship; CIA: Customers' Interest–Attention; ATA: Attitude Towards Advertisement; ATS: Attitude Towards Skin Care Brand; SCPI: Skin Care Product Purchase Intention; WPPP: Willingness to Pay Premium Price.

VIF: Variance Inflation Factors.

**Source:** Authors' data analysis

### 4.5 Multivariate normality

There is no specific requirement of multivariate normality for the partial least squares (PLS) approach, but Peng et al. [93] noted the risk of making a broad generalisation on the capacity of PLS to estimate a model. Therefore, the current study validated the data normality using Web Power online tool (https://webpower.psychstat.org/wiki/tools/index). The results of skewness and kurtosis, as well as the recorded p-values (below the recommended threshold value of 0.05) [94] revealed that the overall data was not normal.

### 4.6 Data analysis

In order to fully comprehend variances among constructs, Hair et al. [92] suggest that variance-based structural equation modelling is helpful when examining the exploratory features and non-normality aspects of the structural model. Partial Least Squares Structural Equation Modelling (PLS-SEM) is a multivariate method that can be used to evaluate the path coefficients between multiple latent variables designed in a complex framework [95]. The major statistical analytic approach for testing the provided hypotheses was variance-based PLS-SEM, which was chosen because the data used in this study are not normal. PLS-SEM was deemed to be the best data analysis approach for the structural model of the current study since it included a large number of components (first and second-order constructs), indicators, and linkages (direct, mediated effects). In light of this, the research utilized Smart-PLS version 3.3.5 as the instrument for carrying out the statistical analysis.

## 5 Results

### 5.1 Demographic characteristics of respondents

Table 2 presents the demographic profile of the respondents. Most of the respondents (55.9%) were female. Besides that, 47.3% of the respondents were of between 21 and 25 years old, and the remaining 41.7% were under 21 years old. The majority of the respondents (78.4%) reported earning monthly income of below RM 2,500, followed by those who reported earning monthly income of between RM 2,501 and RM 5,000 (15.4%). Furthermore, most of the respondents (93.6%) were single, and the remaining 5.4% were married. When it comes to education level, 67.0% of the total respondents attained a Bachelor's degree or equivalent degree, followed by those who completed secondary school (17.3%). Additionally, the respondents were mostly Chinese (82.5%). The majority of the respondents (84.6%) indicated living in the city, whereas the remaining respondents stated living in the rural area.

**Table 2:** Demographic characteristics.

Item	N	%	Item	N	%
*Gender*			*Marital Status*		
Male	343	44.1	Single	728	93.6
Female	435	55.9	Married	42	5.4
Total	778	100.0	Divorced	6	.8
*Age Group*			Widowed	2	.2
			Total	778	100.0
Below 21 years	324	41.7			
21–25 years	368	47.3	*Education*		
26–30 years	34	4.4	Secondary school certificate	135	17.3
31–35 years	19	2.4	Diploma/technical school certificate	111	14.3
36–40 years	10	1.3	Bachelor degree or equivalent	521	67.0
41–45 years	5	.6	Master's degree	6	.8
46–50 years	3	.4	Doctoral degree	5	.6
More than 50 years	15	1.9	Total	778	100.0
Total	778	100.0			
			*Ethnicity*		
*Monthly Income* (RM)			Malay	30	3.8
Below RM2500	610	78.4	Chinese	642	82.5
RM2501-RM5000	120	15.4	Indian	51	6.6
RM5001-RM7500	31	4.0	Others	55	7.1
RM7501-RM10,000	6	.8	Total	778	100.0
RM10,001-RM12500	1	.1			
More than RM12500	10	1.3	*Living Area*		
Total	778	100.0	Urban	658	84.6
			Rural	120	15.4
			Total	778	100.0

**Source:** Authors' data analysis

### 5.2 Reliability and validity

For the current study, Cronbach's alpha and Dijkstra-Hensele's rho were used to determine the reliability of constructs. With values of more than 0.7, all constructs demonstrated excellent reliability. Meanwhile, the average variance extracted (AVE) and composite reliability (CR) were examined to determine the convergent validity of constructs. AVE, with its threshold value of 0.50, serves as the standard indicator of convergent validity or in other words, the extent of variation in the indicators that can be explained by latent variables [96]. As shown in Table 3, all constructs recorded AVE of more than 0.542, suggesting strong convergent validity. The constructs accounted more than 54.2% of total variance in the corresponding indicators. In addition, CR, with its minimum cut-off value of 0.7, is also commonly used as a measure to evaluate internal consistency [95]. All values of CR in Table 3 exceeded 0.842, which reaffirmed high internal consistency of the constructs. Besides that, the Fornell-Larcker criterion was also considered. For this, the square root of AVE values was compared to the latent variable correlations. The square root of the AVE value for each construct must exceed its highest association with any other construct [92]. The results in Table 3 confirmed that all constructs satisfied the Fornell-Larcker criterion.

**Table 3:** Reliability and validity.

Variables	No. Items	Mean	Standard Deviation	Cronbach's Alpha	Dijkstra-Hensele's *rho*	Composite Reliability	Average Variance Extracted
ECT	5	3.345	0.786	0.876	0.879	0.909	0.668
ECP	5	3.892	0.718	0.858	0.863	0.899	0.642
ECD	5	3.322	0.744	0.895	0.904	0.922	0.702
ECE	5	3.278	0.846	0.803	0.817	0.864	0.562
CIC	5	3.293	0.906	0.788	0.792	0.855	0.542
CIA	5	3.397	0.926	0.875	0.878	0.909	0.668
ATA	5	3.284	0.849	0.843	0.843	0.889	0.615
ATS	5	3.438	0.809	0.866	0.871	0.903	0.653
SCPI	5	4.241	1.393	0.930	0.931	0.947	0.781
WPPP	5	3.689	1.341	0.855	0.872	0.897	0.639

**Note:** ECT: Endorsers Credibility–Trustworthiness; ECP: Endorsers Credibility–Exquisite Personality; ECD: Endorsers Credibility–Dignified Image; ECE: Endorsers Credibility–Expertise; CIC: Customers Interest–Companionship; CIA: Customers Interest–Attention; ATA: Attitude Towards Advertisement; ATS: Attitude Towards Skin Care Brand; SCPI: Skin Care Product Purchase Intention; WPPP: Willingness to Pay Premium Price.

**Source:** Authors' data analysis

Adding to that, discriminant validity was determined based on heterotrait-monotrait ratio of correlations (HTMT) [97]. As shown in Table 4, all HTMT values did not exceed the threshold value of 0.85 [98]. Discriminant validity was also determined based on the outcomes of loadings and cross-loadings. The outer-loading of an indicator on the corresponding construct must exceed any of its cross-loadings (correlations) on other constructs in order to determine a model's goodness-of-fit [92]. The recorded loadings and cross-loadings are presented in Appendix 1. All items in this study recorded maximum loadings with the corresponding components, which fulfilled the required criteria.

**Table 4:** Discriminant validity of constructs.

	ECT	ECP	ECD	ECE	CIC	CIA	ATA	ATS	SCPI	WPPP
**Fornell-Larcker Criterion**										
ECT	0.808									
ECP	0.327	0.784								
ECD	0.687	0.420	0.736							
ECE	0.710	0.294	0.673	0.817						
CIC	0.344	0.169	0.338	0.339	0.750					
CIA	0.419	0.275	0.383	0.394	0.190	0.838				
ATA	0.457	0.284	0.465	0.482	0.189	0.388	0.817			
ATS	0.334	0.256	0.365	0.382	0.278	0.197	0.193	0.801		
SCPI	0.598	0.302	0.532	0.582	0.377	0.536	0.546	0.419	0.884	
WPPP	0.595	0.205	0.544	0.592	0.400	0.425	0.508	0.359	0.764	0.800
**Heterotrait-Monotrait Ratio (HTMT)**										
ECT	–									
ECP	0.378	–								
ECD	0.829	0.513	–							
ECE	0.820	0.339	0.806	–						
CIC	0.404	0.218	0.420	0.396	–					
CIA	0.473	0.315	0.452	0.442	0.229	–				
ATA	0.521	0.327	0.558	0.549	0.226	0.422	–			
ATS	0.386	0.299	0.443	0.437	0.327	0.222	0.221	–		
SCPI	0.667	0.338	0.620	0.643	0.431	0.584	0.602	0.466	–	
WPPP	0.689	0.235	0.660	0.679	0.470	0.478	0.584	0.410	0.848	–

**Note:** ECT: Endorsers Credibility–Trustworthiness; ECP: Endorsers Credibility–Exquisite Personality; ECD: Endorsers Credibility–Dignified Image; ECE: Endorsers Credibility–Expertise; CIC: Customers Interest–Companionship; CIA: Customers Interest–Attention; ATA: Attitude Towards Advertisement; ATS: Attitude Towards Skin Care Brand; SCPI: Skin Care Product Purchase Intention; WPPP: Willingness to Pay Premium Price.

**Source:** Authors' data analysis

### 5.3 Path analysis

Referring to the first part of the results in Table 5 on the correlations of all credibility attributes of celebrity endorsers with ATA, ECT (*β* = 0.100, *p* = 0.026), ECP (*β* = 0.075, *p* = 0.028), ECD (*β* = 0.152, *p* = 0.001), and ECE (*β* = 0.221, *p* = 0.000) recorded positive beta coefficients and statistically significant p-values, which confirmed the significant and positive influence of all four attributes on ATA. Thus, H1a, H2a, H3a, and H4a were accepted. Meanwhile, CIC recorded a negative path coefficient (−0.019) and a non-significant p-value (0.310), which suggested its significant influence on ATA. In other words, H5a was rejected. On the other hand, CIA recorded a positive path coefficient (0.184) and a significant p-value (0.000), which indicated its significant and positive influence on ATA. Thus, H6a was supported.

**Table 5:** Path analysis.

Hypo		Beta	CI-Min	CI-Max	*T*	*p*	*R*^2^	*f*^*2*^	*Q*^*2*^	Decision
H1a	ECT→ATA	0.100	0.015	0.180	1.949	0.026	0.309	0.006	0.203	Accepted
H2a	ECP→ATA	0.075	0.011	0.134	1.920	0.028		0.007		Accepted
H3a	ECD→ATA	0.152	0.068	0.228	3.298	0.001		0.014		Accepted
H4a	ECE→ATA	0.221	0.139	0.305	4.542	0.000		0.030		Accepted
H5a	CIC→ATA	−0.019	−0.081	0.047	0.495	0.310		0.000		Rejected
H6a	CIA→ATA	0.184	0.117	0.254	4.264	0.000		0.039		Accepted
H1b	ECT→ATS	0.021	−0.085	0.114	0.342	0.366	0.191	0.000	0.122	Rejected
H2b	ECP→ATS	0.116	0.045	0.171	2.862	0.002		0.013		Accepted
H3b	ECD→ATS	0.112	0.023	0.197	2.121	0.017		0.007		Accepted
H4b	ECE→ATS	0.207	0.107	0.290	3.862	0.000		0.023		Accepted
H5b	CIC→ATS	0.142	0.063	0.211	3.004	0.001		0.021		Accepted
H6b	CIA→ATS	0.005	−0.078	0.079	0.102	0.459		0.000		Rejected
H7	ATA→SCPI	0.484	0.410	0.541	11.998	0.000	0.399	0.376	0.311	Accepted
H8	ATS→SCPI	0.326	0.267	0.402	8.070	0.000		0.171		Accepted
H9	SCPI→WPPP	0.764	0.728	0.793	39.262	0.000	0.582	1.398	0.366	Accepted

**Note:** ECT: Endorsers Credibility–Trustworthiness; ECP: Endorsers Credibility–Exquisite Personality; ECD: Endorsers Credibility–Dignified Image; ECE: Endorsers Credibility–Expertise; CIC: Customers Interest–Companionship; CIA: Customers Interest–Attention; ATA: Attitude Towards Advertisement; ATS: Attitude Towards Skin Care Brand; SCPI: Skin Care Product Purchase Intention; WPPP: Willingness to Pay Premium Price.

**Source:** Authors' data analysis

The second part of the results in Table 5 on the correlations of all credibility attributes of celebrity endorsers with ATS revealed positive beta coefficients and statistically significant p-values of ECP (*β* = 0.116, *p* = 0.002), ECD (*β* = 0.112, *p* = 0.017), and ECE (*β* = 0.207, *p* = 0.000). These results confirmed the significant and positive influence of these three attributes on ATS. Thus, H2b, H3b, and H4b were supported. On the other hand, ECT recorded a non-significant p-value of 0.366, which implied its insignificant influence on ATS. Therefore, H1b was rejected. Meanwhile, CIC (*β* = 0.142, *p* = 0.001) was found to contribute significant influence on ATS. In other words, H5b was accepted. CIA recorded a non-significant p-value of 0.459, which indicated its insignificant influence on ATS. Thus, H6b was rejected.

The third part of the results in Table 5 on the correlations of ATA and ATS with SCPI revealed that both ATA (*β* = 0.848, *p* = 0.000) and ATS (*β* = 0.326, *p* = 0.000) recorded positive beta coefficients and statistically significant p-values. These results revealed the significant and positive effects of ATA and ATS on SCPI. Thus, both H7 and H8 were supported.

Referring to the final part of the results in Table 5 on the relationship between SCPI and WPPP, SCPI recorded a positive path coefficient of 0.764 and a significant p-value (0.000), which indicated the significant influence of SCPI on WPPP. Thus, H9 was supported.

Adding to that, the predictive power of the study's model was examined based on the coefficient of determination (*R*^*2*^), specifically the amount of variance in endogenous constructs explained by all associated exogenous constructs [92]. Endogenous latent variables with *R*^*2*^ values of 0.75, 0.50, or 0.25 can be categorised as substantial, moderate, or weak, respectively [92]. As for the current study, the obtained results revealed low to moderate variance explained: ATA with *R*^*2*^ of 0.309; ATS with *R*^*2*^ of 0.191; SCPI with *R*^*2*^ of 0.399; WPPP with *R*^*2*^ of 0.582.

Apart from the *R*^*2*^ values, *Q*^*2*^ values should also be examined to ensure the predictive accuracy of the model [92]. *Q*^*2*^ values of more than zero in a structural model for a certain reflective endogenous latent variable indicate the predictive relevance of the model for the dependent variable [92]. As shown in Table 5, *Q*^*2*^ values for ATA (0.203), ATS (0.122), SCPI (0.311), and WPPP (0.366) exceeded zero, which indicated the significant predictive relevance of all components.

### 5.4 Mediating effects

Referring to Table 6, the obtained results demonstrated that ATA significantly and positively mediated the relationships of ECT (*β* = 0.048, *p* = 0.033), ECP (*β* = 0.036, *p* = 0.033), ECD (*β* = 0.074, *p* = 0.001), ECE (*β* = 0.107, *p* = 0.000), CIA (*β* = 0.089, *p* = 0.000) with SCPI. The results further revealed that ATA demonstrated no significant mediating effect on the relationship between CIC and SCPI (*β* = - 0.009, *p* = 0.496).

**Table 6:** Mediating effects.

Associations	Beta	CI -Min	CI -Max	*t*	*p*	Decision
ECT→ATA→SCPI	0.048	0.004	0.088	1.845	0.033	Partial Mediation
ECP→ATA→SCPI	0.036	0.005	0.068	1.837	0.033	Partial Mediation
ECD→ATA→SCPI	0.074	0.033	0.111	3.243	0.001	Partial Mediation
ECE→ATA→SCPI	0.107	0.066	0.152	4.252	0.000	Partial Mediation
CIC→ATA→SCPI	−0.009	−0.038	0.024	0.496	0.310	No Mediation
CIA→ATA→SCPI	0.089	0.053	0.138	3.554	0.000	Partial Mediation
ECT→ATS→SCPI	0.007	−0.027	0.039	0.337	0.368	No Mediation
ECP→ATS→SCPI	0.038	0.016	0.058	2.818	0.003	Partial Mediation
ECD→ATS→SCPI	0.037	0.008	0.066	2.116	0.017	Partial Mediation
ECE→ATS→SCPI	0.068	0.037	0.103	3.447	0.000	Partial Mediation
CIC→ATS→SCPI	0.046	0.021	0.078	2.649	0.004	Partial Mediation
CIA→ATS→SCPI	0.002	−0.024	0.026	0.101	0.460	No Mediation
ATA→SCPI→WPPP	0.369	0.312	0.423	10.827	0.000	Partial Mediation
ATS→SCPI→WPPP	0.249	0.203	0.305	7.811	0.000	Partial Mediation

**Note:** ECT: Endorsers Credibility–Trustworthiness; ECP: Endorsers Credibility–Exquisite Personality; ECD: Endorsers Credibility–Dignified Image; ECE: Endorsers Credibility–Expertise; CIC: Customers Interest–Companionship; CIA: Customers Interest–Attention; ATA: Attitude Towards Advertisement; ATS: Attitude Towards Skin Care Brand; SCPI: Skin Care Product Purchase Intention; WPPP: Willingness to Pay Premium Price.

**Source:** Authors' data analysis

Besides that, this study examined the mediating effects of ATS. The obtained results confirmed that ATS significantly and positively mediated the relationships of ECP (*β* = 0.038, *p* = 0.003), ECD (*β* = 0.037, *p* = 0.017), ECE (*β* = 0.068, *p* = 0.000), CIC (*β* = 0.046, *p* = 0.004) with SCPI. The results further demonstrated that ATA did not mediate the relationships of ECT (*β* = 0.007, *p* = 0.368) and CIC (*β* = 0.002, *p* = 0.460) with SCPI. The results in Table 6 also demonstrated the significant and positive mediating effects of SCPI on the relationships of ATA (*β* = 0.369, *p* = 0.000) and ATS (*β* = 0.249, *p* = 0.000) with WPPP.

## 6 Discussion

The current study highlighted the significance of credibility attributes of celebrity endorsers (trustworthiness, exquisite personality, dignified image, and expertise) on Malaysian consumers’ purchasing behaviour for green skincare products during COVID-19. Overall, Malaysian consumers tend to demonstrate favourable attitude towards green skincare product advertisements and attitude towards green skincare product brands in the case of high credibility attributes of celebrity endorsers, resulting in higher purchase intention and willingness to pay premium price for green skincare products. According to the demographic profile of the respondents, a large percentage of the participants are between the ages of 18 and 25. In this regard, we deduce that customers aged 18 and up have sufficient access to money for purchasing skin care products, as these products do not cost a lot of money to obtain. Furthermore, according to recent survey reports by Statista, consumers aged 18 to 29 are likely to spend more money than any other age group on skin care, beauty, and personal care products in the United States, China, and many other Asian nations [7,99]. In light of the statistical information, it can be concluded that the sample in this study can be regarded as being generalized.

As a first observation, the analysis showed that the credibility, exquisite personality, dignified image, and knowledge of celebrity endorsers significantly and positively influenced Malaysian consumers' opinions towards commercials for green skincare products. Previous research on the impact of celebrity endorsement has found similar results [17,18,24,25]. That is to say, when celebrities recommend any product, it may help the brand attract new customers quickly when the celebrities present themselves as having honesty and reliability. The possible explanation is that the beautiful and elegant appearance, a respectable and positive off-screen personality, and relevant experience of celebrities make advertisements more compelling and appealing to consumers.

This study revealed a substantial correlation between consumers' attention and attitude towards advertisements for green skincare products, which is supported by the findings revealed in the research of Ding et al. [38]. The outcome may indicate that advertising may appeal to consumers if it features consumers' favourite celebrities, and consumers prefer celebrities to support the product they are considering purchasing. Nonetheless, the association between customer companionship and attitude towards green skincare brand advertisements was found to be minor, contradicting the outcomes of few earlier researches [23,38]. The possible explanation for this result is that customers might have more product alternatives, more product varieties, and more offers with advanced product features while viewing a lot of online advertisements during the COVID-19 lockdown period. Another plausible reason is that consumers were more observant and preferred checking in on the latest trends to getting engaged in celebrities’ activities. However, celebrities often promote numerous brands of similar products at a time, resulting in a lack of focus among their fans and possibly confusing buyers when choosing a product. Another possible explanation is that brands or products advised by customers' acquaintances or peers appear to be more dependable and useful than brands or products promoted by celebrities. Because of fans' favouritism for celebrities, many viewers of advertisements starring celebrity endorsers may pay close attention and show interest; nevertheless, this interest may not always reflect on actual sales of the endorsed product or brand.

Next in the analysis, the results showed that the attitude of consumers towards green skincare brands was highly influenced by the exquisite personalities, dignified images, and expertise of celebrity endorsers. These findings corroborated earlier studies showing that customers choose a brand more favourably when it is recommended by a prominent public figure [16,17,20,24]. The likely reason is that brands gain a wealthy and elegant impression when consumers observe that they can afford high-profile celebrities to support their brands. In a more thorough explanation, it may be stated that Malaysian consumers have a favourable impression towards green skincare brands that are promoted by celebrities because they believe that the celebrities' involvement secures the brands' foundation of Malaysian cultural, social, and religious values.

Notably, the study result revealed that customers' attitudes towards green skincare brands and their purchase intentions were negligibly impacted by the trustworthiness of celebrity endorsers. Earlier research findings in a similar context contradicted these results [16,21,22]. The possible reason can be stated: the trustworthiness of celebrity endorsers may highly impact consumers' attitudes, mostly towards the brands that are extensively popular or well-established in the market and towards the brands that are frequently used in B&PC. Since the demand for green beauty products and brands has just recently begun to rise, consumers are yet to trust those brands. Hence, despite the celebrities' support, the green skincare brands may fail to gain credibility among customers. Another likely reason is that Malaysian celebrities are yet to get extensive expertise on green skincare products, based on which they may be considered trustworthy when endorsing a brand. On the basis of probable reasoning and judgements regarding the trustworthiness of celebrities and brands, this study thus posits that consumers' likelihood to choose a certain brand may increase if a highly credible celebrity endorses that brand.

Companionship was found to have an insignificant association with consumers’ attitudes towards green skincare product brands. This may be attributed to the COVID-19 lockdown period, when consumers did not have access to these skincare products at the physical stores and could not test them on the spot. As a result, when it comes to online purchases, consumers may be hesitant to buy green skincare products without first testing them, even if these brands have been endorsed by their favourite celebrities. The result indicates that despite customers' acknowledgment that their favourite celebrities are highly recommending the green skin care products brand, they are not yet ready to use those. This gap between companionship and attitude may widen due to other factors such as a lack of detailed product knowledge.

The results of the current study also revealed the positive and significant influence of consumers' attitude towards green skincare product advertisements and attitude towards green skincare product brands on their purchase intention. These results were found to be in line with the findings of numerous prior studies [16,17,27,28,66,82]. Moreover, purchase intention was found to significantly and positively mediate the effects of consumers' attitude towards green skincare product advertisements and attitude towards green skincare product brands on their willingness to pay premium price. These results supported the findings of several recent studies [31,53] on the positive influence of a brand having a celebrity endorser with credible and charming personality on consumers’ attitude toward the endorsed brand and willingness to pay for the brand.

## 7 Implications

### 7.1 Theoretical implications

Through the integration of two prominent theories, specifically the S–O-R theory and PSI, this study presented a holistic framework on the significance of celebrity endorsement in marketing and promotion strategies for green skincare products during COVID-19 crisis. The framework was found to be an excellent fit for the current study's purpose of advancing the relevant theories and literature on celebrity endorsement. Prior studies mainly focused on celebrity attributes like attractiveness, skills, and trustworthiness. Referring to the recommendation by Singh and Banerjee [82], this study expanded the models proposed by earlier studies by incorporating dignified image as another significant credibility attributes of celebrity endorsers. With respect to the PSI theory, the current study examined the influence of two key components of consumers' interests, namely companionship and attention, as recommended by Ding et al. [38] and Cheah et al. [23]. The current study presented critical findings on the significance of celebrity endorsers as well as valuable empirical evidence on the impact of celebrity endorsements on consumers' involvement in celebrity-endorsed advertisements and brands. The study's findings have enhanced our knowledge of celebrity endorsers' credibility features such as personality, dignity, image, and expertise, from which marketing literature can benefit from the perspective of theory implementation in market segments and innovative marketing practices.

### 7.2 Practical implications

Apart from the study's theoretical contributions on the advantages of having celebrity endorsers as a significant marketing strategy to stimulate consumers' purchase intention and willingness to pay premium price, this study offered several practical implications. The significant shift in customer purchasing behaviour during the COVID-19 crisis emphasises the urgent necessity to deliver more convincing and compelling content about a brand's products. Consumers are driven to online shopping, particularly during the COVID-19 lockdown period, making them more dependent on celebrity endorsements, their reviews, and their trustworthiness.

The findings of the current study substantially benefit cosmetics industry players and other relevant stakeholders in determining how advertisements involving celebrity endorsers and celebrity-endorsed brands influence consumers’ purchasing decisions [14]. According to the findings of the study, celebrities' exquisite personalities have a substantial influence on customers' attitudes toward advertising, purchase intentions, and willingness to pay a premium price for green skincare products. This implies that marketers should be especially cautious when selecting celebrity endorsers, paying close attention to how well celebrities' personalities fit with the features of the products they are promoting.

Besides that, celebrity endorsers' dignified image was found to have substantial influence on consumers' attitude towards advertisements and attitude towards green skincare product brands. Advertisers should capitalise on this relationship by appointing a noble celebrity with an uncontroversial lifestyle. This finding shows that marketers should also pay attention to celebrity endorsers' off-screen lifestyles in order to identify the ideal celebrity to successfully portray their product while aligning with the celebrities' healthy lifestyle values. Customers may perceive an advertisement featuring a celebrity endorser as a publicity hoax if they have a negative image of the celebrity endorser's off-screen unhealthy lifestyle.

Despite the fact that the study found no significant influence of celebrity trustworthiness, it does suggest that brands should delegate celebrities who are not only well-known or popular, but also have a track record of widespread consumer acceptance for what they endorsed in the past. Alternatively, because this study found that celebrities' expertise has a strong influence, marketers should take special care in selecting celebrities with extensive knowledge and prior experience in green cosmetic products. This finding suggests that celebrities' trustworthiness among consumers can be boosted by their knowledge and skill levels.

Furthermore, the current study revealed the significance of consumers' companionship and attention for celebrities as another important factor that enhances consumers’ attitudes towards advertisements and green skincare product brands. As a consequence, marketing professionals should assess the intensity of consumers' attachment to celebrities. For example, celebrity endorsers with a large number of followers, reviews, and comments clearly promote the endorsed product or brand because they are accompanied by seamless customer favouritism. Likewise, marketers should identify which celebrities are receiving the most attention so that this attention may be exploited to earn consumers' favourable attitudes, purchase intentions, and willingness to pay a premium price for green skincare products. This is because celebrities who have a lot of followers and a large number of fans may easily raise environmental awareness and the significance of green and healthy cosmetics among consumers.

## 8 Conclusion

The global production and marketing of green skincare products have recently gained growing interest. The involvement of celebrity endorsers stimulates favourable perceptions of the endorsed brands and products among consumers, which are expected to drive consumers' purchase intention and even willingness to pay premium price. Focusing on green skincare products, the current study incorporated two prominent theories, namely the S–O-R theory and PSI, empirically examined the influence of credibility attributes of celebrity endorsers (specifically trustworthiness, exquisite personality, dignified image, and expertise) and consumers’ interests (specifically attention and companionship) on purchase intention and willingness to pay premium price among Malaysian consumers during COVID-19.

Based on the current study's findings, the credibility attributes of celebrity endorsers and consumers' interests have substantial effects on consumers' attitude towards green skincare product advertisements and attitude towards green skincare product brands, which subsequently influence their purchase intention. As a result, customers' purchase intentions enhanced their decision to spend more on green skin care products. Besides, consumers' attitudes towards celebrity endorsements of green skincare product brands and advertisements have a large mediating influence on their willingness to pay a premium price. This study thus emphasised the importance of appointing celebrity endorsers with strong credibility traits who can precisely convey the appropriate message to consumers of green beauty and personal care products. This is because consumers' confidence in celebrity endorsements would strengthen their willingness to pay a premium price for the eco-friendly product, despite its lack of a strong market presence. Consequently, this would encourage marketers and product designers to develop more socially acceptable and environmentally friendly commodities.

The current study developed a comprehensive framework on the relevance of celebrity endorsers' credibility and useful insights on consumers' interest in celebrity endorsements, both of which are becoming incredibly influential as a marketing tactic. This study's findings presented a substantial benefit for academics and practitioners in the marketing segment to effectively utilise the credibility features of celebrity endorsers and consumers' interests, with the aim of promoting a stronger emphasis on gaining more profitability through the premium pricing method. Since the notion of shopping has evolved heavily toward online purchases, incorporating a range of credibility qualities of celebrity endorsers, all types of green product sectors and industries may benefit from celebrities'.

Despite these notable findings, the current study encountered several limitations. Firstly, the study's model and findings cannot be generalized due to the small sample size and limited timeframe. Secondly, the study focused on the young consumers which may restrict the generalizability of the consumers age group. It is recommended for future research to consider a larger sample size with numerous demographic population and varied contexts for improving generalizability. Furthermore, the study's model can be explored with various product categories endorsed by various celebrities. Secondly, the potential negative implications of celebrity-endorsed advertisements were not considered in the current study. Therefore, it is recommended for future research to consider the boundary conditions that influence the perceptions of consumers on celebrities and the endorsed brands in the cases of low and high credibility. Lastly, the current study focused on a specific setting. Addressing this, it is recommended for future research to consider brand comparisons and consumers' perceptions of both local and global celebrities.

## Ethical approval

Human Research Ethics Committee of Changzhi University approved this study (CZ-202300050).

## Author(s) contribution

*Abdullah Al Mamun*: Performed the experiments; Analyzed and interpreted the data; Contributed reagents, materials, analysis tools or data; Wrote the paper. *Farzana Naznen*: Conceived and designed the experiments; Performed the experiments; Wrote the paper. *Qing Yang*: Conceived and designed the experiments; Performed the experiments; Wrote the paper. *Mohd Helmi Ali*: Conceived and designed the experiments; Performed the experiments; Wrote the paper. *Nik Mohd Hazrul Nik Hashim*: Conceived and designed the experiments; Analyzed and interpreted the data; Wrote the paper. All authors approved the final version of the manuscript and give their consent for submission and publication.

## Availability of data and materials

The original contributions presented in the study are included in the article/Supplementary Material (**S2.** Dataset), further inquiries can be directed to the corresponding author/s.

## Funding

This research received no specific grant from any funding agency in the public, commercial, or not-for-profit sectors.

## Declaration of competing interest

The authors declare that they have no known competing financial interests or personal relationships that could have appeared to influence the work reported in this paper.

## Figures and Tables

**Table:** 

Code	ECT	ECP	ECD	ECE	CIC	CIA	ATA	ATS	SCPI	WPPP
ECT1	*0.877*	0.288	0.593	0.599	0.329	0.362	0.404	0.298	0.521	0.537
ECT2	*0.851*	0.220	0.563	0.540	0.306	0.330	0.374	0.278	0.482	0.513
ECT3	*0.776*	0.224	0.499	0.638	0.248	0.308	0.330	0.227	0.464	0.442
ECT4	*0.768*	0.302	0.519	0.548	0.219	0.367	0.385	0.277	0.473	0.439
ECT5	*0.760*	0.283	0.597	0.555	0.285	0.323	0.343	0.262	0.474	0.464
ECP1	0.222	*0.775*	0.290	0.204	0.169	0.174	0.185	0.225	0.202	0.142
ECP2	0.238	*0.832*	0.310	0.213	0.137	0.216	0.209	0.233	0.224	0.158
ECP3	0.243	*0.809*	0.286	0.204	0.103	0.212	0.199	0.164	0.218	0.124
ECP4	0.259	*0.804*	0.337	0.221	0.131	0.270	0.260	0.147	0.240	0.154
ECP5	0.306	*0.695*	0.403	0.295	0.117	0.202	0.249	0.222	0.287	0.211
ECD1	0.375	0.429	*0.656*	0.364	0.181	0.229	0.297	0.260	0.324	0.282
ECD2	0.517	0.423	*0.753*	0.463	0.238	0.312	0.350	0.278	0.392	0.367
ECD3	0.512	0.162	*0.711*	0.525	0.277	0.245	0.341	0.236	0.392	0.487
ECD4	0.527	0.270	*0.772*	0.538	0.290	0.318	0.357	0.276	0.435	0.446
ECD5	0.584	0.273	*0.783*	0.574	0.255	0.297	0.364	0.292	0.410	0.417
ECE1	0.615	0.235	0.556	*0.829*	0.275	0.326	0.408	0.295	0.484	0.467
ECE2	0.556	0.296	0.579	*0.813*	0.287	0.301	0.392	0.338	0.483	0.486
ECE3	0.583	0.171	0.554	*0.838*	0.298	0.319	0.418	0.310	0.499	0.559
ECE4	0.594	0.249	0.559	*0.833*	0.287	0.362	0.393	0.343	0.487	0.466
ECE5	0.555	0.253	0.497	*0.771*	0.230	0.300	0.356	0.269	0.417	0.437
CIC1	0.315	0.150	0.301	0.319	*0.818*	0.142	0.151	0.221	0.302	0.306
CIC2	0.346	0.089	0.324	0.344	*0.795*	0.140	0.160	0.238	0.366	0.440
CIC3	0.182	0.162	0.214	0.191	*0.624*	0.159	0.162	0.112	0.212	0.186
CIC4	0.249	0.017	0.237	0.231	*0.802*	0.097	0.125	0.229	0.266	0.345
CIC5	0.171	0.238	0.176	0.160	*0.689*	0.186	0.120	0.218	0.247	0.179
CIA1	0.335	0.260	0.307	0.292	0.165	*0.827*	0.244	0.151	0.420	0.307
CIA2	0.359	0.238	0.336	0.344	0.190	*0.851*	0.303	0.180	0.441	0.361
CIA3	0.376	0.232	0.333	0.355	0.202	*0.855*	0.349	0.177	0.496	0.388
CIA4	0.337	0.173	0.306	0.334	0.127	*0.844*	0.281	0.169	0.436	0.357
CIA5	0.345	0.245	0.317	0.318	0.114	*0.813*	0.409	0.149	0.441	0.356
ATA1	0.385	0.227	0.391	0.408	0.145	0.362	*0.854*	0.184	0.475	0.443
ATA2	0.381	0.296	0.401	0.408	0.179	0.374	*0.821*	0.133	0.473	0.413
ATA3	0.316	0.282	0.342	0.361	0.121	0.267	*0.816*	0.080	0.383	0.319
ATA4	0.398	0.197	0.384	0.389	0.200	0.337	*0.818*	0.214	0.478	0.434
ATA5	0.380	0.155	0.377	0.402	0.119	0.227	*0.775*	0.168	0.410	0.456
ATS1	0.267	0.308	0.286	0.268	0.137	0.190	0.218	*0.672*	0.298	0.186
ATS2	0.261	0.180	0.302	0.323	0.280	0.164	0.164	*0.808*	0.344	0.318
ATS3	0.252	0.164	0.288	0.304	0.257	0.125	0.108	*0.857*	0.305	0.288
ATS4	0.262	0.176	0.261	0.283	0.225	0.101	0.077	*0.827*	0.309	0.299
ATS5	0.292	0.203	0.317	0.340	0.206	0.201	0.196	*0.830*	0.407	0.330
SCPI1	0.553	0.305	0.504	0.521	0.335	0.479	0.479	0.414	*0.881*	0.660
SCPI2	0.538	0.307	0.465	0.486	0.328	0.511	0.504	0.388	*0.884*	0.625
SCPI3	0.522	0.237	0.491	0.522	0.351	0.479	0.496	0.349	*0.896*	0.719
SCPI4	0.531	0.230	0.469	0.523	0.353	0.463	0.470	0.375	*0.899*	0.711
SCPI5	0.499	0.259	0.420	0.517	0.298	0.435	0.466	0.326	*0.858*	0.655
WPPP1	0.495	0.263	0.466	0.496	0.306	0.360	0.403	0.278	*0.658*	0.754
WPPP2	0.512	0.158	0.465	0.523	0.373	0.352	0.425	0.353	0.673	*0.874*
WPPP3	0.477	0.123	0.440	0.469	0.328	0.329	0.418	0.291	0.586	*0.819*
WPPP4	0.491	0.140	0.448	0.501	0.316	0.392	0.441	0.300	0.657	*0.888*
WPPP5	0.389	0.125	0.340	0.355	0.268	0.247	0.334	0.187	0.437	*0.636*

**Note:** ECT: Endorsers Credibility–Trustworthiness; ECP: Endorsers Credibility–Exquisite Personality; ECD: Endorsers Credibility–Dignified Image; ECE: Endorsers Credibility–Expertise; CIC: Customers Interest–Companionship; CIA: Customers Interest–Attention; ATA: Attitude Towards Advertisement; ATS: Attitude Towards Skin Care Brand; SCPI: Skin Care Product Purchase Intention; WPPP: Willingness to Pay Premium Price.

**Source:** Authors' data analysis
