# Supplementary material for: Modelling the significance of celebrity endorsement and consumer interest on attitude, purchase intention, and willingness to pay a premium price for green skincare products
Source: Heliyon. 2023 May 26;9(6):e16765. doi: 10.1016/j.heliyon.2023.e16765 (PMC10245062; doi:10.1016/j.heliyon.2023.e16765)
Supplement: Multimedia component 1 [file mmc1.docx]

**Supplementary Material S1.** Survey Instrument

**Endorsers Credibility** - **Trustworthiness**

1. Celebrities are trustworthy.
2. Celebrities are reliable.
3. Celebrities are knowledgeable about the product and/or brand they endorse.
4. Celebrities are compatible with the product that they endorse.
5. Celebrities are ethical.

**Endorsers Credibility** - **Exquisite Personality**

1. Celebrities are handsome/beautiful.
2. Celebrities are physically attractive.
3. Celebrities are glamorous.
4. Celebrities are charming.
5. Celebrities are sophisticated.

**Endorsers Credibility** - **Dignified Image**

1. Celebrities make contribution for social causes.
2. Celebrities show empathy through their helpful actions in time of natural disaster.
3. Celebrities are not involved in any controversy.
4. Celebrities speak up for issues of national importance.
5. Celebrities are humble.

**Endorsers Credibility** - **Expertise**

1. Celebrities knows a lot about the product
2. Celebrities are competent to make assertions about the product
3. Celebrities are expert on the product
4. Celebrities sufficiently experienced to make assertions about the product
5. Celebrities are aware about the effects of the product

**Customers Interest - Companionship**

1. I look forward to watch celebrity’s recent advertisement/endorsement
2. If celebrities switch to another green skin care brand, I would use green skin care products as well
3. If celebrities advertise/endorse any new products, I would check the details about that product
4. I miss seeing them when they do not advertise/endorse products
5. I would like to meet them in person

**Customers Interest - Attention**

1. Advertisement with celebrity endorsement attracts me
2. Advertisement with celebrity endorsement draws my full attention
3. Advertisement with celebrity endorsement makes me interested in green skin care products
4. Advertisement with celebrity endorsement makes me like green skin care products more
5. Advertisement with celebrity endorsement gives me a good impression of the green skin care products

**Attitude towards Advertisement**

1. I feel the advertisement with celebrity endorsement was very good
2. The advertisement with celebrity endorsement was enjoyable
3. The advertisement with celebrity endorsement was interesting
4. There was a lot about the advertisement with celebrity endorsement that I liked
5. The advertisement with celebrity endorsement was believable

**Attitude towards Skin Care Brand**

1. The advertisement with celebrity endorsement gave a positive impression of the brand
2. Based on the advertisement with celebrity endorsement, I like the brand
3. Based on the advertisement with celebrity endorsement, I think the brand is very good
4. Based on the advertisement with celebrity endorsement, I think the brand is very useful
5. Based on the advertisement with celebrity endorsement, my opinion of the brand is very favorable

**Skin Care Product Purchase Intention**

1. I’m likely to purchase green skin care products endorsed by celebrities
2. I’m interested in the green skin care products endorsed by celebrities
3. I plan on buying green skin care products endorsed by celebrities
4. I intend to try green skin care products endorsed by celebrities
5. I am interested in tasting green skin care products endorsed by celebrities

**Willingness to pay premium price**

1. The price of green skin care products endorsed by celebrities would have to increase quite a bit before I would switch to another brand.
2. I am willing to pay a higher price for green skin care products endorsed by celebrities than for other brands.
3. I avoid buying green skin care products not endorsed by celebrities
4. I am willing to pay a lot more for green skin care products endorsed by celebrities than for other brands.
5. I am willing to pay ___% more for green skin care products endorsed by celebrities over other brands: 0% ꞁ 5% ꞁ 10% ꞁ 15% ꞁ 20% and more
